# Timing of total joint arthroplasty post-COVID-19: an evaluation of the optimal window to minimize perioperative risks

**DOI:** 10.1186/s42836-024-00275-x

**Published:** 2024-10-04

**Authors:** Henry Hoang, Beshoy Gabriel, Brandon Lung, Steven Yang, Justin P. Chan, the N3C Consortium

**Affiliations:** Department of Orthopaedic Surgery, University of California Irvine, Orange, CA 92868 USA

**Keywords:** Total hip arthroplasty, Total knee arthroplasty, COVID-19, Charlson Comorbidity Index, Postoperative complications, Venous thromboembolism

## Abstract

**Background:**

Total hip arthroplasty (THA) and total knee arthroplasty (TKA) are commonly performed orthopedic procedures. This study aimed to evaluate the impact of COVID-19 status on postoperative complications and mortality in patients undergoing THA and TKA.

**Methods:**

A total of 110,186 underwent either THA or TKA. Patients were grouped based on their COVID-19 status, gathered from the National COVID-19 Cohort Collaborative (N3C) in the 12 weeks preceding surgery and compared for various variables, including age, sex, BMI, and Charlson Comorbidity Index (CCI) scores. COVID-19 status was defined as a positive test result that was closest to the date of surgery regardless of testing positive previously. Postoperative complications such as venous thromboembolism (VTE), sepsis, surgical site infection, bleeding, acute kidney injury (AKI), 30-day, and 1-year all-cause mortality were examined. To compare the variables, an odds ratio with a 95% confidence interval was calculated with a significant level set at *P* < 0.05. Logistic regression using R programming was utilized for these calculations.

**Results:**

Univariate analysis was performed and rates of VTE (1.02% vs. 3.35%), 30-day mortality (0.25% vs. less than 5%), and 1-year mortality (1.42% vs. 5.43%) were higher in the COVID-19-positive group for THA patients (*P* < 0.001). For TKA patients, only 30-day mortality was significantly higher in the COVID-19-positive group (*P* = 0.034). Multivariate logistic regression revealed that a positive COVID-19 diagnosis within two weeks of surgery and a CCI score > 3 were significant predictors of postoperative complications and mortality for both TKA and THA.

**Conclusions:**

Patients with a positive COVID-19 diagnosis within 12 weeks of THA or TKA carried a significantly higher risk for postoperative complications and mortality. In addition, a CCI score > 3 is also a significant risk factor. These findings emphasize the importance of vigilant preoperative screening and risk stratification in the era of COVID-19.

**Supplementary Information:**

The online version contains supplementary material available at 10.1186/s42836-024-00275-x.

## Introduction

The COVID-19 pandemic has led to a global health crisis of unprecedented magnitude, exerting a profound impact on the realm of orthopedic surgery. In particular, orthopedic surgery, as a field, has faced considerable disruptions in recent years due to the dynamic interplay between administering timely surgical interventions and the demands of appropriately managing COVID-19 cases [1, 2]. This is particularly relevant in hip and knee arthroplasty cases where the risks and clinical outcomes could potentially be influenced by COVID-19.

The American Joint Replacement Registry found that 2.2 million hip and knee arthroplasties were done between 2012 and 2020, and the majority were primary total knee arthroplasties (TKA) (54.5%) and primary total hip arthroplasties (THA) (38.6%) [3]. Given the significant amount of patients who elect to undergo TKA or THA, it is crucial to investigate the impact of COVID-19 prior to surgery on post-surgical outcomes to best guide orthopedic surgeons on when it is optimal to operate. After all, it is now well-known that the presence of COVID-19 compromises the immune system, causing patients to be more susceptible to infections while also hindering wound healing [4]. Additionally, recent research has found that respiratory issues associated with COVID-19 could significantly increase the chances of postoperative complications, ranging from pneumonia or acute respiratory distress syndrome to death [5–8]. This could presumably lead to impediments to not only rehabilitation but also functional recovery following the procedures.

However, apart from the inherent risks associated with THA or TKA, both the surgical procedures and COVID-19 independently induce a hypercoagulable state. Patients undergoing total hip and knee arthroplasty are already at a high risk for venous thromboembolism (VTE), such as deep vein thrombosis (DVT), with an incidence of approximately 0.6–1.5% [9]. Concurrently, COVID-19 infection has also been linked to a hypercoagulable state, increasing the risk of thrombosis and pulmonary embolism [2, 10, 11]. It is possible that when combined with COVID-19-induced hypercoagulability, the risk for thrombotic complications in THA and TKA becomes even more pronounced. This effect can result in a higher incidence of blood clot formation during the postoperative period. VTE and DVT present significant risks to patients undergoing THA and TKA, posing potential serious complications and adverse impacts on patient outcomes [12].

The primary objective of this study was to investigate if COVID-19 infection prior to THA or TKA is associated with an increased risk of postoperative complications. We hypothesized that patients with surgery closest to the date of their COVID-19 diagnosis would have the highest complication rates. While existing literature details outcomes following arthroplasty in COVID-19 positive patients, our research utilized a comprehensive database to not only assess postoperative outcomes, but also determine the optimal timing for surgery based on the timing of their COVID-19 diagnosis.

## Materials and methods

The National COVID-19 Cohort Collaborative (N3C) is a research initiative established by the NIH to address critical research questions related to the COVID-19 pandemic. With a database encompassing 20.1 million cases, N3C constitutes one of the largest and most diverse COVID-19 patient cohorts globally. It serves as a centralized data platform that integrates electronic health records from multiple institutions, offering access to extensive clinical and research data on COVID-19 patients. Standardization procedures are applied to the data to ensure consistency across multiple institutions. Moreover, the accuracy and completeness of the data are upheld through rigorous quality assurance measures implemented by committees such as the N3C data harmonization team [13].

We conducted a retrospective cohort study using data collected from the National COVID-19 Cohort Collaborative between 15 March 2020, and April 2023 from major academic and community institutions. COVID-19 positivity was identified by using the ICD-10 code U07.1, which provides a timestamp denoting the day of COVID-19 diagnosis. It is imperative to acknowledge that while this ICD-10 code indicates the date of COVID-19 reactivity, it does not precisely denote the onset of infection. Consequently, our study primarily focused on the period during which patients tested positive for COVID-19 rather than the onset of symptoms. The study included participants of all ages, and the COVID-19-positive cohort was compared with COVID-19 negative controls in terms of age, sex, race, Charlson Comorbidity Scores, BMI, and vaccination status. Notably, no exclusion criteria were applied based on primary diagnosis (e.g., arthritis, fracture, tumor, or other conditions). Comorbidity data were collected using ICD-10 codes mapped to Systemic Nomenclature of Medicine (SNOMED) codes for comprehensive assessment and analysis.

### Querying process

To establish the cohorts, we employed specific Current Procedural Terminology (CPT) codes. THA included CPT codes 27125, 27130, 27132, 27134, 27137, and 27138, while TKA involved 27445, 27447, 27486, and 27487 (Supplementary Table S1). Patients were included in the COVID-19-positive group if they had a positive COVID-19 diagnosis within the 12 weeks preceding their surgery. This cohort was further stratified based on the timing of their COVID-19 diagnosis: 0–2 weeks before surgery, 2–6 weeks before surgery, and 6–12 weeks before surgery. After identifying the COVID-19-positive and COVID-19-negative cohorts who underwent hip or knee arthroplasty, we used ICD-10 codes (Supplementary Table S2) to query for conditions such as pulmonary thrombosis, sepsis, acute kidney injury (AKI), surgical site infection, 30-day mortality, and one-year mortality.

### Analysis variables

In our study, the primary independent variable was COVID-19 positivity, operationally defined as individuals who received a positive COVID-19 diagnosis within the 12 weeks before their arthroplasty surgery. We also analyzed several patient characteristics, including age, race, gender, body mass index (BMI), smoking status, diabetes, and hypertension. Our study outcomes consisted of 30-day mortality, 1-year mortality, VTE, sepsis, AKI, and surgical site infection. These postoperative complications occurred within 90 days of surgery (besides the 1-year mortality).

### Statistical analysis

We conducted descriptive statistics to evaluate baseline patient characteristics and factors. Continuous variables were expressed as mean ± SD. Categorical variables were compared using odds ratios with a 95% confidence interval to assess the strength of associations. Statistical significance was defined as a *P* < 0.05.

Multivariate logistic regression using standard R packages was employed for statistical analysis. The “glm” function (generalized linear model) was utilized to investigate the relationship between a positive COVID-19 diagnosis in the 0–2 weeks or 2–6 weeks before surgery, and CCI score > 3 for postoperative complications of DVT, as well as, 30-day and 1-year all-cause mortality.

## Results

**Fig. 1:**
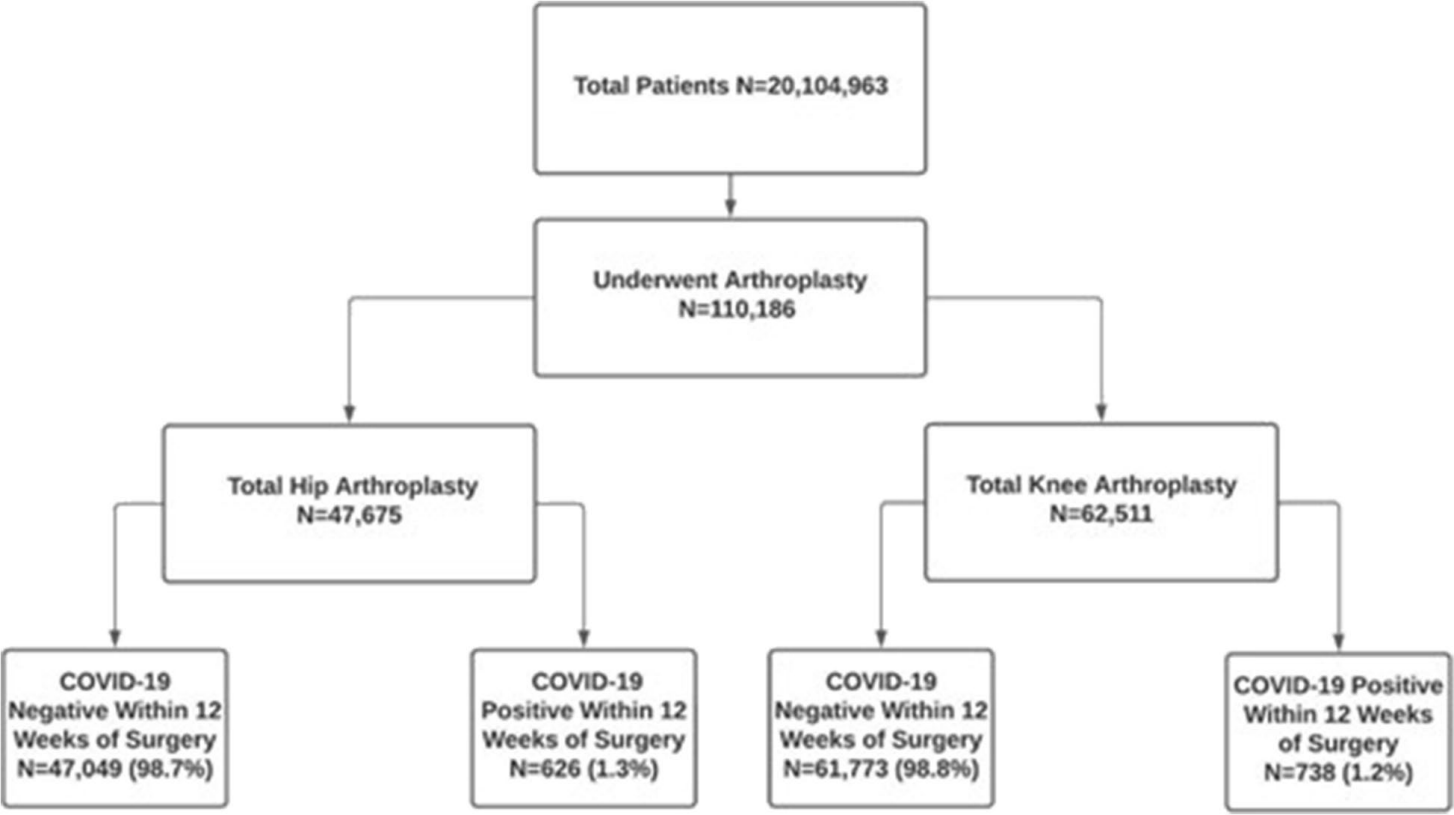
Flowchart of study querying

A total of 20,104,963 patients were queried and 110,186 underwent either total knee or hip arthroplasty. 47,675 patients underwent THA and 62,511 received TKA. The two groups both comprised patients who were COVID-19-positive and those who were not. In the THA group, 98.7% of patients were negative for COVID-19 in the 12 weeks preceding surgery while the remaining 1.3% were positive in the 12 weeks prior to surgery. Similarly, in the TKA group, 98.8% of patients were negative in the 12 weeks before surgery with the remaining 1.2% positive in the preoperative 12 weeks (Fig. 1).

When comparing patients undergoing THA and TKA without and with a COVID-19 diagnosis within 12 weeks prior to surgery, there were significant differences in age (68.03 ± 10.65 vs. 67.17 ± 10.72, *P* = 0.003) and sex distribution (41.4% male vs. 45.0% male, 58.6% female vs. 55.0% female, *P* = 0.007) between the two groups. BMI (33.95 ± 7.37 vs. 33.62 ± 7.71, *P* = 0.099) and Charlson Comorbidity Index (CCI) scores (2.47 ± 2.71 vs 2.48 ± 2.74, *P* = 0.85) were comparable between patients with a positive COVID-19 diagnosis and those without (Table 1). Postoperative complications included VTE or DVT, sepsis, surgical site infection, bleeding, AKI, 30-day all-cause mortality, and 1-year all-cause mortality.

**Table 1:** All Hip and Knee Arthroplasty Demographics. Comparison of patient demographics and medical comorbidities between COVID-19-positive and COVID-19-negative cohorts. Values are reported as *n* (%) unless otherwise specified

Characteristic	No COVID diagnosis within 12 weeks of Surgery (*n* = 108,822)	COVID diagnosis within 12 weeks of Surgery (*n* = 1364)	*P*
Age (y), (mean ± SD)	68.03 (10.65)	67.17 (10.72)	0.003
Sex, *n* (%)			0.007
Male	41.4%	45.0%	
Female	58.6%	55.0%	
Ethnicity, *n* (%)			0.041
White	88,722 (81.5%)	1104 (80.9%)	
Black or African American	14,303 (13.1%)	196 (14.3%)	
Asian	1651 (1.51%)	≤ 20	
BMI, (mean ± SD)	33.95 (7.37)	33.62 (7.71)	0.099
Vaccine Doses Before Surgery			< 0.001
0 COVID Vaccine Doses Before Surgery	83,408 (76.6%)	896 (65.7%)	
1 COVID Vaccine Dose Before Surgery	1660 (1.52%)	45 (3.39%)	
2 COVID Vaccine Doses Before Surgery	6908 (6.34%)	92 (6.74%)	
3 COVID Vaccine Doses Before Surgery	4370 (4.01%)	91 (6.67%)	
≥ 4 COVID Vaccine Doses Before Surgery	9084 (8.34%)	213 (15.61%)	
Charlson Comorbidity Index, (mean ± SD)	2.47 (2.71)	2.48 (2.74)	0.850

For patients that underwent THA, the rates of VTE (1.02% vs. 3.35%, *P* < 0.001), 30-day mortality (0.25% vs. less than 5%*, *P* < 0.001), and 1-year mortality (1.42% vs. 5.43%, *P* < 0.001) were higher in the COVID-19-positive group than in the COVID-19-negative group (Table 2). For patients who received TKA, the rates of 30-day mortality (0.09% vs. less than 3%*, *P* = 0.034) were higher in the COVID-19-positive group than in the COVID-19-negative group (Table 3). The N3C requires that any category with less than 20 patients be obfuscated to protect patient privacy.

**Table 2:** Clinical Outcomes of Total Hip Arthroplasty. Comparison of perioperative complication rates between COVID-19-positive and COVID-19-negative cohorts. Values are reported as *n* (%)

Clinical Outcome (*Occurring Within 90 days of Surgery)	No COVID Diagnosis within 12 weeks of Surgery (*n* = 47,049)	COVID diagnosis within 12 weeks of Surgery (*n* = 626)	*P*
VTE	482 (1.02%)	21 (3.35%)	< 0.001
Sepsis	462 (0.98%)	≤ 20	0.22
Surgical Site Infection	621 (1.32%)	≤ 20	0.48
Bleeding	22 (0.04%)	≤ 20	0.26
AKI	63 (0.13%)	≤ 20	0.57
30-Day Mortality	121 (0.25%)	≤ 20	< 0.001
365-Day Mortality	670 (1.42%)	34 (5.43%)	< 0.001

**Table 3:** Clinical Outcomes of Total Knee Arthroplasty. Comparison of perioperative complication rates between COVID-19-positive and COVID-19-negative cohorts. Values are reported as *n* (%)

Clinical Outcome (Occurring Within 90 days of Surgery)	No COVID Diagnosis within 12 weeks of Surgery (*n* = 61,773)	COVID Diagnosis within 12 weeks of Surgery (*n* = 738)	*P*
VTE	816 (0.01)	≤ 20	0.327
Sepsis	424 (0.007)	≤ 20	1
Surgical Site Infection	656 (0.01)	≤ 20	1
Bleeding	< 20	0	1
AKI	74 (0.001)	0	1
30-Day Mortality	57 (0.0009)	≤ 20	0.034
365-Day Mortality	383 (0.0006)	≤ 20	0.054

**Fig. 2:**
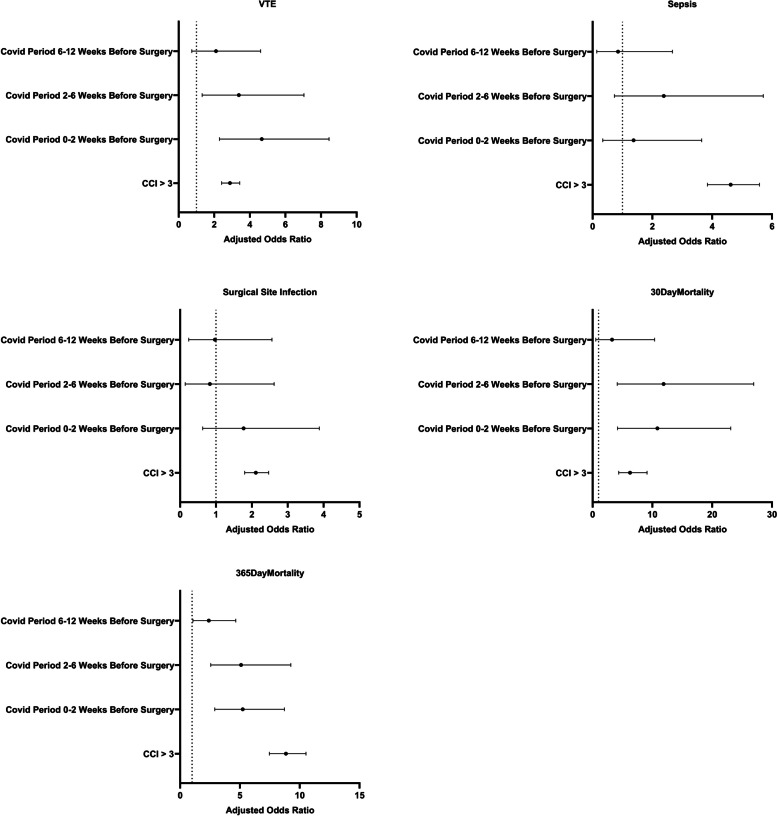
Odds Ratios for postoperative complications in THA patients with a confirmed COVID-19 diagnosis compared to controls without a positive COVID-19 test for the 84 days prior to surgery. Error bars represent a 95% confidence interval

On multivariate logistic regression for patients who underwent THA, a positive COVID-19 diagnosis in the 0–2 weeks or 2–6 weeks prior to surgery, and CCI score > 3 were found to be significant predictors of DVT, 30-day, and 1-year all-cause mortality. After adjusting for COVID-19 status prior to THA and CCI score > 3, positive COVID-19 status in the 2 weeks preceding surgery carried a significantly increased risk for DVT (Odds Ratio 4.67, 95% CI [2.29, 8.45]), 30-day mortality (OR 10.83, 95% CI [4.17, 23.11]), and 1-year mortality (OR 5.24, 95% CI [2.90, 8.73]). Similarly, positive COVID-19 status between 2–6 weeks prior to THA surgery posed a significantly increased risk for DVT (OR 3.38, 95% CI [1.32, 7.04]), 30-day mortality (OR 11.88, 95% CI [4.13, 26.95]), and 1-year mortality (OR 5.10, 95% CI [2.56, 9.26]). CCI score > 3 resulted in an increased risk for DVT (OR 2.88, 95% CI [2.41, 3.43]), sepsis (OR 4.62, 95% CI [3.84, 5.58]), surgical site infection (OR 2.11, 95% CI [1.80, 2.47]), AKI (OR 5.70, 95% CI [3.72, 10.03]), 30-day mortality (OR 6.26, 95% CI [4.37, 9.11]), and 1-year mortality (OR 8.85, 95% CI [7.46, 10.53]) (Table 4 and Fig. 2).

**Table 4:** Association between COVID-19 and risk of perioperative complications in THA patients. Values are reported as Odds Ratio (95% Confidence Interval)

Characteristic	**Odds Ratio**	**Adjusted Odds Ratio**	***P***
*DVT*			
CCI > 3	2.53 [2.11, 3.04]	2.88 [2.41, 3.43]	< 0.001
COVID Period 0–2 Weeks Before Surgery	5.01 [2.46, 9.10]	4.67 [2.29, 8.45]	< 0.001
COVID Period 2–6 Weeks Before Surgery	3.49 [1.36, 7.30]	3.38 [1.32, 7.04]	0.004
COVID Period 6-12Weeks Before Surgery	2.31 [0.82, 5.09]	2.10 [0.74, 4.61]	0.104
*Sepsis*			
CCI > 3	4.37 [3.61, 5.32]	4.62 [3.84, 5.58]	< 0.001
COVID Period 0–2 Weeks Before Surgery	1.45 [0.36, 3.86]	1.37 [0.34, 3.65]	0.587
COVID Period 2–6 Weeks Before Surgery	2.41 [0.74, 5.78]	2.38 [0.73, 5.71]	0.089
COVID Period 6–12 Weeks Before Surgery	0.91 [0.15, 2.86]	0.85 [0.14, 2.67]	0.816
*Surgical Site Infection*			
CCI > 3	2.08 [1.77, 2.46]	2.11 [1.80, 2.47]	< 0.001
COVID Period 0–2 Weeks Before Surgery	1.99 [0.70, 4.38]	1.77 [0.63, 3.88]	< 0.001
COVID Period 2–6 Weeks Before Surgery	0.86 [0.14, 2.70]	0.83 [0.14, 2.62]	0.004
COVID Period 6–12 Weeks Before Surgery	0.97 [0.24, 2.57]	0.97 [0.24, 2.56]	0.104
*Acute Kidney Injury*			
CCI > 3	5.70 [3.34, 10.04]	5.70 [3.72, 10.03]	< 0.001
COVID Period 0–2 Weeks Before Surgery	N/A	N/A	
COVID Period 2–6 Weeks Before Surgery	N/A	N/A	
COVID Period 6–12 Weeks Before Surgery	N/A	N/A	
*30-Day All-Cause Mortality*			
CCI > 3	6.25 [4.28, 9.28]	6.26 [4.37, 9.11]	< 0.001
COVID Period 0–2 Weeks Before Surgery	9.03 [3.43, 19.65]	10.83 [4.17, 23.11]	< 0.001
COVID Period 2–6 Weeks Before Surgery	13.21 [4.56, 30.33]	11.88 [4.13, 26.95]	< 0.001
COVID Period 6–12 Weeks Before Surgery	3.48 [0.57, 11.23]	3.25 [0.53, 10.37]	0.101
*365-Day All-Cause Mortality*			
CCI > 3	8.96 [7.50, 10.76]	8.85 [7.46, 10.53]	< 0.001
COVID Period 0–2 Weeks Before Surgery	4.91 [2.70, 8.34]	5.24 [2.90, 8.73]	< 0.001
COVID Period 2–6 Weeks Before Surgery	5.47 [2.72, 9.95]	5.10 [2.56, 9.26]	< 0.001
COVID Period 6–12 Weeks Before Surgery	2.60 [1.15, 5.06]	2.40 [1.07, 4.65]	0.018

**Fig. 3:**
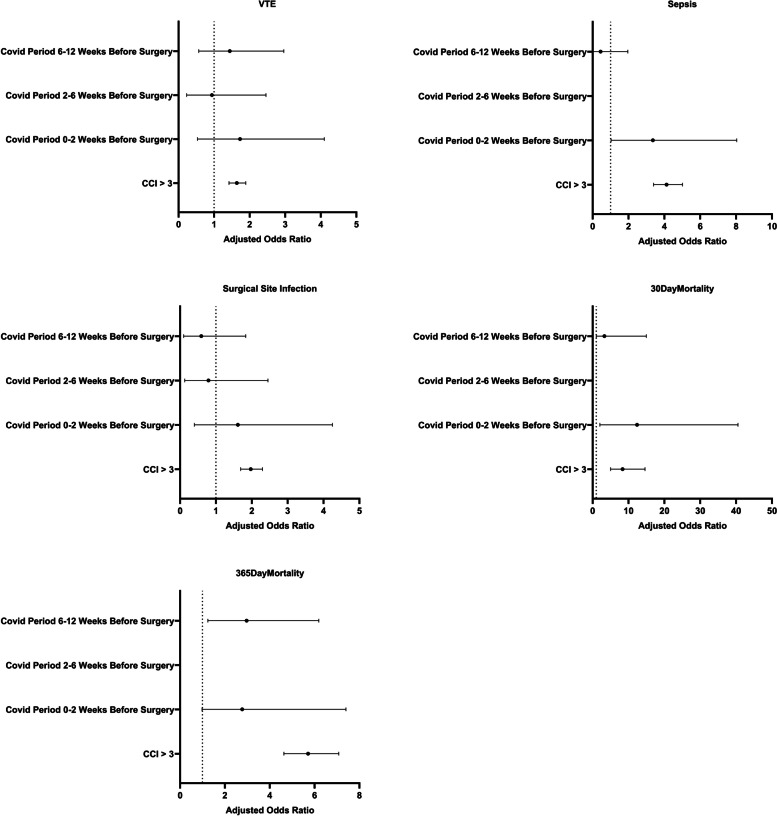
Odds Ratios for postoperative complications in TKA patients with a confirmed COVID-19 diagnosis compared to controls without a positive COVID-19 test for the 84 days prior to surgery. Error bars represent a 95% confidence interval

For patients receiving TKA, multivariate logistic regression demonstrated that a positive COVID-19 diagnosis in the 0–2 weeks before surgery and a CCI score > 3 were found to be significant predictors of sepsis and 30-day mortality. When adjusting for COVID-19 status prior to TKA surgery and a CCI score > 3, positive COVID-19 status in the 2 weeks preceding surgery presented a significantly increased risk for sepsis (OR 3.36, 95% CI [1.03, 8.04]) and 30-day mortality (OR 12.39, 95% CI [2.01, 40.52]). A CCI score > 3 resulted in a significantly increased risk for DVT (OR 1.64, 95% CI [1.42, 1.89]), sepsis (OR 4.12, 95% CI [3.40, 5.01]), surgical site infection (OR 1.97, 95% CI [1.69, 2.30]), 30-day mortality (OR 8.35, 95% CI [5.01, 14.60]), and 1-year mortality (OR 5.71, 95% CI [4.63, 7.08]) (Table 5 and Fig. 3).

**Table 5:** Association between COVID-19 and risks of perioperative complications in TKA patients. Values are reported as Odds Ratio (95% Confidence Interval)

Characteristic	**Odds Ratio**	**Adjusted Odds Ratio**	***P***
*DVT*			
CCI > 3	1.54 [1.33, 1.78]	1.64 [1.42, 1.89]	< 0.001
COVID Period 0–2 Weeks Before Surgery	1.75 [0.54, 4.14]	1.73 [0.53, 4.10]	0.279
COVID Period 2–6 Weeks Before Surgery	0.95 [0.24, 2.50]	0.94 [0.23, 2.46]	0.910
COVID Period 6-12Weeks Before Surgery	1.48 [0.58, 3.04]	1.44 [0.57, 2.96]	0.379
*Sepsis*			
CCI > 3	3.98 [3.25, 4.87]	4.12 [3.40, 5.01]	< 0.001
COVID Period 0–2 Weeks Before Surgery	3.39 [1.03, 8.12]	3.36 [1.03, 8.04]	0.018
COVID Period 2–6 Weeks Before Surgery	N/A	N/A	0.959
COVID Period 6–12 Weeks Before Surgery	1.00 [0.03, 2.06]	0.44 [0.03, 1.96]	0.414
*Surgical Site Infection*			
CCI > 3	2.08 [1.77, 2.44]	1.97 [1.69, 2.30]	< 0.001
COVID Period 0–2 Weeks Before Surgery	.60 [0.40, 4.24]	1.61 [0.40, 4.25]	0.416
COVID Period 2–6 Weeks Before Surgery	0.81 [0.13, 2.52]	0.79 [0.13, 2.45]	0.734
COVID Period 6–12 Weeks Before Surgery	0.59 [0.10, 1.84]	0.59 [0.10, 1.83]	0.452
*Acute Kidney Injury*			
CCI > 3			
COVID Period 0–2 Weeks Before Surgery	N/A	N/A	
COVID Period 2–6 Weeks Before Surgery	N/A	N/A	
COVID Period 6–12 Weeks Before Surgery	N/A	N/A	
*30-Day All-Cause Mortality*			
CI > 3	4.64 [2.67, 8.32]	8.35 [5.01, 14.60]	< 0.001
COVID Period 0–2 Weeks Before Surgery	12.65 [2.05, 41.65]	12.39 [2.01, 40.52]	< 0.001
COVID Period 2–6 Weeks Before Surgery	N/A	N/A	0.977
COVID Period 6–12 Weeks Before Surgery	3.05 [0.17, 14.23]	3.27 [0.99, 14.96]	0.242
*365-Day All-Cause Mortality*			
CCI > 3	5.01 [4.02, 6.26]	5.71 [4.63, 7.08]	< 0.001
COVID Period 0–2 Weeks Before Surgery	2.83 [0.69, 7.60]	2.77 [0.98, 7.40]	0.008
COVID Period 2–6 Weeks Before Surgery	N/A	N/A	
COVID Period 6–12 Weeks Before Surgery	3.04 [1.19, 6.34]	2.97 [1.24, 6.19]	0.009

Interestingly, a positive COVID-19 status between 6–12 weeks prior to surgery also significantly increased the risk of 1-year mortality for both THA (OR 2.40, 95% CI [1.07, 4.65]) and TKA (OR 2.97, 95% CI [1.24, 6.19]).

## Discussion

Our analysis demonstrated that COVID-19 prior to arthroplasty was associated with an increased risk of severe postoperative complications. Indeed, we did observe that patients receiving surgery closest to the date of their COVID-19 diagnosis had the highest complication rates (Tables 4 & 5). We found that a positive COVID-19 diagnosis in the 6 weeks prior to THA was associated with significantly increased DVT risk, 30-day and 1-year all-cause mortality. A positive COVID-19 diagnosis in the 2 weeks before TKA did not yield better outcomes in terms of significantly increased rates of sepsis and 30-day all-cause mortality. Although not particularly significant, it should still be noted that there was a possibility that 1-year mortality was associated as well (OR 2.77, 95% CI [0.98, 7.40]. Other studies in the literature suggested a similar trend in mortality. A previous study by Nahshon et al. found that the postoperative mortality rate due to severe pulmonic complications in patients with COVID-19 during the perioperative period was 27.5% [5, 14]. Another study by Maniscalco et al. found a significant increase in the number of postoperative deaths in the 3 weeks following surgical treatment of proximal femur fractures with either THA, HA, or other techniques, primarily due to cardio-respiratory arrest [15].

To help decide on when it is the best time to operate or how long to postpone an operation, it is important to understand the relationship between the time of COVID-19 diagnosis and surgery in general. In a recent study by the COVIDSurg Collaborative, the authors found that 30-day mortality diminished when undergoing surgery 7 weeks or more after being infected with COVID-19. Similarly, Butyrskii et al. concluded that surgeries after 7–8 weeks had the same 30-day mortality rate as in patients without infection. These findings are echoed by the current American Society of Anesthesiologists guidelines, which recommends anywhere from a 4–12 week surgery delay following COVID-19 infection, depending on the severity of the patient’s infection, vaccination status, and other comorbidities [16]. However, these guidelines are not specific to THA or TKA procedures.

Recent research has focused on examining the connection between COVID-19 infections and the outcomes of total joint arthroplasty (TJA). In a case–control study conducted by Bae et al., no notable differences were observed in postoperative complications, such as DVT, wound dehiscence, or peri-prosthetic joint infection between patients who had COVID-19 within one week after surgery and those who did not [17]. While this finding contrasts with our data, potential reasons could include the time frame of Bae et al.’s study, which spanned from 2021 to 2022. This period omitted the early years of the pandemic when vaccination campaigns were still in their infancy. Additionally, their study excluded patients with multiple comorbidities, which could also account for the discrepancy. A multicenter retrospective analysis by Clement et al. found no significant differences in mortality rates among patients who underwent elective TJA and subsequently tested positive for COVID-19 [12]. This is in contrast to our study, which indicated a noteworthy mortality rate. It's important to highlight that Clement et al. defined a COVID-19 positive case based on either a positive antigen or PCR test. However, their research, which spanned the year 2020, was conducted during a time when comprehensive and widespread COVID-19 testing was still in its early stages. Consequently, out of 1073 patients in their study, only 5 were identified as COVID-19 positive, a surprisingly low number given the circumstances.

A comprehensive retrospective cohort study by Heckmann et al. focused on the varying rates of postoperative complications in patients who had been diagnosed with COVID-19 within a 90-day window surrounding their THA or TKA procedures [18]. The study found that patients who tested positive for COVID-19 within 90 days before or after their surgery developed significantly more complications. Specifically, they had a 4.23-fold increased risk of pulmonary embolism (PE), a 2.75-fold heightened risk of wound dehiscence, a 4.8-fold greater risk of acute respiratory failure, and a staggering 12.69-fold elevated risk of pneumonia among total knee replacement patients. Similarly, for THA patients, the risks were substantial, with an 8.77-fold increased risk of PE, a 9.58-fold greater risk of prosthetic joint infection (PJI), a 3.47-fold heightened risk of wound dehiscence, a 4.56-fold increased risk of acute respiratory failure, and an astonishing 16.81-fold elevated risk of pneumonia. These findings underscore the vulnerability of COVID-19-positive individuals in the peri-operative period, emphasizing the significantly heightened susceptibility to postoperative complications. Importantly, it is worth noting that the study primarily covered the year 2020, a time when the severity of COVID-19 was at its peak, and widespread vaccination was not as readily available. Consequently, the observed severe postoperative complications may be attributable to the unique challenges posed by the pandemic during that period.

Efforts have previously been made to discern the temporal relationship between a COVID-19 diagnosis and postoperative complication rates in patients undergoing TJA. Notably, studies by Forlenza et al. and Lung et al. provided valuable insights [19, 20]. In their retrospective cohort analysis, Forlenza et al. established a clear link between a COVID-19 diagnosis and increased postoperative risks. Their data indicated a marked rise in the odds of developing complications such as DVT (OR 4.86, 95% CI 2.10–11.81, *P* < 001), pulmonary embolism (OR 6.27, 95% CI 2.57–16.71, *P* < 001), and other postoperative issues (OR 3.36, 95% CI 2.47–4.59, *P* < 001). Intriguingly, the risk of these complications was acutely heightened when the surgical procedure was performed closer to the COVID-19 diagnosis, particularly within the first three months. This emphasized the profound influence of COVID-19 on postoperative outcomes and reiterated the temporal nature of this relationship. However, our findings diverge in certain areas, potentially due to variations in the study time-frames.

Lung et al. previously showed that patients who contracted COVID-19 prior to undergoing elective THA or TKA did not exhibit a significant uptick in complications such as respiratory, infectious, cardiac, or thromboembolic events up to six months post-surgery. It is worth noting that procedures for patients with a prior COVID-19 diagnosis were deferred for at least 4 weeks, ensuring a negative test result before surgery. This approach corroborates our conclusion of a temporal link between COVID-19 diagnosis and post-operative complication rates and suggests evidence for an optimal time window of surgical postponement to reduce the incidence of postoperative complications.

First, COVID-19 studies have found that many patients may continue to have COVID-19 symptoms more than 2 to 3 months after the acute illness, commonly referred to as long COVID [21, 22]. Second, a host of cardiovascular abnormalities has been reported among patients beyond the acute phase, ranging from myocardial inflammation, myocardial infarction, right ventricular dysfunction, and arrhythmias [22]. Third, COVID-19 seems to be associated with a hypercoagulable state, which can substantially increase the risk for VTE and DVT [4, 10, 23]. As such, it may be inappropriate to simply use the length of time from the initial illness to determine when to perform THA or TKA.

Furthermore, the hospital environment poses an increased risk of acquiring secondary infections, adding to the complexity and risk of the postoperative period [24]. As such, healthcare providers must carefully evaluate the risks, test for COVID-19 prior to surgery, and closely monitor patients to mitigate the impact of COVID-19 on surgical outcomes. Further protocols should be formulated to enhance patient safety and ensure optimal timing of surgical interventions after COVID-19 infection while taking into account the inherently increased risk for DVT in both COVID-19 and arthroplasty procedures. Given the findings presented in this study, it may even be warranted to postpone non-urgent hip surgeries for more than 12 weeks following a COVID-19 infection or to consider a more aggressive VTE chemoprophylaxis regimen for urgent THA in COVID-19 patients. In light of this, developing a preoperative scoring system to evaluate the risk of specific postoperative complications for patients undergoing THA or TKA after a recent COVID-19 infection would be prudent. Such a tool could evaluate COVID-19 infection timing, the patient’s risk for VTE or DVT, and other patient comorbidities.

It is also important to acknowledge the limitations of our study. The N3C database does not contain information on the severity of COVID-19 infection outcomes. Although we were able to control for the effect of preoperative comorbidities, which might make a person more susceptible to the effect of COVID-19, it is possible that the severity of infection may have had a direct effect on the postoperative complication profile. Our study focused on the period after a patient first tested positive for COVID-19, which likely encompasses a wide clinical spectrum of symptomatology, ranging from asymptomatic infection to severe respiratory failure. With the present data, we were also not able to determine the effects of vaccination status on complications since a large portion of the data consists of patients from the early phases of the pandemic. It is possible that, with vaccinations, the passage of time, and resultant herd immunity, some of the noted complication risks may eventually become less pronounced. Lastly, given the retrospective design of this study, we are only able to show an association but not causation between preoperative COVID-19 infection and the negative postoperative outcomes outlined in this study.

## Conclusions

In conclusion, our study underscored the importance of considering the timing of COVID-19 diagnosis in relation to the scheduled date of THA or TKA. Patients who elect to undergo THA or TKA within 6 weeks of their COVID-19 diagnosis may be at increased risk for DVT, sepsis, and death within a month. Additionally, given that our findings demonstrated that COVID-19 up to 12 weeks before surgery was associated with significantly increased 1-year all-cause mortality for both THA and TKA, it is imperative for further research to focus on the longer-term impacts and mortality of COVID-19 prior to arthroplasty procedures.

## Supplementary Information

Additional file 1: Supplementary Table S1. CPT codes for Total Hip and Total Knee Arthroplasty.

Additional file 2: Supplementary Table S2. ICD10 codes for Post-Operative Complications.

## Data Availability

The dataset supporting the conclusions of this article is available in the NIH N3C repository: https://ncats.nih.gov/research/research-activities/n3c
