# Supplementary material for: Timing of total joint arthroplasty post-COVID-19: an evaluation of the optimal window to minimize perioperative risks
Source: Arthroplasty. 2024 Oct 4;6:53. doi: 10.1186/s42836-024-00275-x (PMC11452997; doi:10.1186/s42836-024-00275-x)
Supplement: Supplementary file 1 — Additional file 1: Supplementary Table S1. CPT codes for Total Hip and Total Knee Arthroplasty. [file 42836_2024_275_MOESM1_ESM.docx]

| **Hip Arthroplasty Surgery** | 27125,27130,27132,27134,27137,27138 |
| --- | --- |

| **Total Knee Arthroplasty** | 27445,27447,27486,27487 |
| --- | --- |

**Supplementary Table 1: CPT codes for Total Hip and Total Knee Arthroplasty**
